# Depressive Symptoms and Cardiovascular Health by the American Heart Association’s Definition in the Reasons for Geographic and Racial Differences in Stroke (REGARDS) Study

**DOI:** 10.1371/journal.pone.0052771

**Published:** 2012-12-26

**Authors:** Ian M. Kronish, April P. Carson, Karina W. Davidson, Paul Muntner, Monika M. Safford

**Affiliations:** 1 Center for Behavioral Cardiovascular Health, Columbia University, New York, New York, United States of America; 2 Department of Epidemiology, University of Alabama at Birmingham, Birmingham, Alabama, United States of America; 3 Division of Preventive Medicine, University of Alabama at Birmingham, Birmingham, Alabama, United States of America; Pennington Biomedical Research Center, United States of America

## Abstract

**Background:**

Depressive symptoms are associated with increased incident and recurrent cardiovascular events. In 2010, the American Heart Association published the Life’s Simple 7, a metric for assessing cardiovascular health as measured by 4 health behaviors (smoking, physical activity, body mass index, diet) and 3 biological measures (cholesterol, blood pressure, glucose). The association between depressive symptoms and the Life’s Simple 7 has not yet been explored.

**Methods:**

Data from 20,093 participants ≥45 years of age who enrolled in the Reasons for Geographic and Racial Differences in Stroke (REGARDS) study between 2003 and 2007 and who had complete data available on Life’s Simple 7 components were used for these analyses. The prevalence of ideal, intermediate, and poor health on each Life’s Simple 7 component and total Life’s Simple 7 scores were compared between participants with and without depressive symptoms. Depressive symptoms were measured using the 4-item Centers for Epidemiologic Studies of Depression scale.

**Results:**

Participants with depressive symptoms were more likely to have poor levels on each of the Life’s Simple 7 components other than cholesterol [adjusted prevalence ratios (95% CI): smoking 1.41 (1.29–1.55); physical activity 1.38 (1.31–1.46); body mass index 1.09 (1.04–1.15); diet 1.08 (1.06–1.10); blood pressure 1.11 (1.02–1.21); glucose 1.24 (1.09–1.41)]. There was a graded association between increasing depressive symptoms and lower total Life’s Simple 7 score.

**Conclusion:**

Depressive symptoms are associated with worse cardiovascular health on the overall Life’s Simple 7 and on individual components representing both health behaviors and biological factors.

## Introduction

Individuals with elevated depressive symptoms, irrespective of whether the symptoms meet criteria for a clinical diagnosis of major depression, are at increased risk for incident and recurrent cardiovascular events.[1]–[5] The increased cardiovascular risk attributable to depressive symptoms may result from unfavorable cardiovascular health behaviors or shared biological mechanisms linking depression and cardiovascular disease. [6] While prior studies have shown that patients with depressive symptoms are less likely to perform certain cardiovascular health behaviors,[7]–[9] studies of the association between depressive symptoms and health behaviors have been limited by focusing on only one health behavior (e.g., smoking alone [10]), utilizing non-validated questions to assess behavior (e.g., “Are you following a heart healthy diet?” [8]), or being restricted to certain populations such as post-menopausal women. [5] The association between depressive symptoms and biological measures like blood pressure and cholesterol is even less clear with some studies finding associations[11]–[16], but not others.[17]–[19].

In 2010, the American Heart Association (AHA) published a metric for assessing overall cardiovascular health in adults. [20](See Table 1) This metric is comprised of 4 health behaviors (cigarette smoking, physical activity, body mass index, and diet) and 3 biological measures (cholesterol, blood pressure, blood sugar) of cardiovascular health. These 7 components have been labeled Life’s Simple 7 for public health messaging. According to the metric, persons with all of these factors in the ideal range are classified as having “ideal” cardiovascular health, and others are classified as having “intermediate” or “poor” cardiovascular health depending on whether they have any components in the intermediate or poor levels. Although this metric was derived according to a consensus of experts, a lower number of ideal components on this metric has subsequently been associated with a higher incidence of cardiovascular disease and higher mortality. [21]–[22].

**Table 1:** Definitions of the American Heart Association’s Life’s Simple 7 Components^a.^

Life’s Simple 7 component	Definition
Smoking	
Ideal	Never or quit >12 months
Intermediate	Former, quit ≤12 months
Poor	Current
Body Mass Index	
Ideal	<25 kg/m^2^
Intermediate	25–29.99 kg/m^2^
Poor	≥30 kg/m^2^
Physical activity^b^	
Ideal	≥4 times/week
Intermediate	1–3 times/week
Poor	none
Healthy diet^c^	
Ideal	4–5 healthy diet criteria
Intermediate	2–3 healthy diet criteria
Poor	0–1 healthy diet criteria
Total cholesterol	
Ideal	<200 mg/dl, without medication
Intermediate	200–239 mg/dl or treated to <200 mg/dl
Poor	≥240 mg/dl
Blood pressure	
Ideal	SBP/DBP<120/<80 mmHg, without medication
Intermediate	SBP of 120–139 or DBP 80–89 mmHg or treated to SBP/DBP<120/<80 mmHg
Poor	SBP≥140 or DBP≥90 mmHg
Fasting serum glucose^d^	
Ideal	<100 mg/dl, without medication
Intermediate	100–125 mg/dl or treated to <100 mg/dl
Poor	≥126 mg/dl

Abbreviations: SBP, systolic blood pressure; DBP, diastolic blood pressure.

a As per Lloyd-Jones et al [20].

b Definitions of ideal and intermediate levels of physical activity were modified from Lloyd-Jones et al [20] in which ideal level was defined as ≥150 minutes/week of moderate intensity activity or ≥75 minutes/week of vigorous activity and intermediate level was defined as 1–149 minutes/week of moderate intensity activity or 1–74 minutes/week of vigorous intensity activity.

c Healthy diet criteria included: 1) fish consumption ≥2 servings/week, 2) fruits or vegetables ≥4.5 cups/day, 3) sodium intake <1500 mg/day, 4) sugar <450 kcal/week, and 5) fiber/carbohydrate ratio >0.1.

d To avoid excluding non-fasting participants, the following cut-points were used to classify the glucose component in participants (n = 2,378) who did not fast prior to their study visit: ideal <140 mg/dl; intermediate 140–199 mg/dl or treated to <140 mg/dl; poor ≥200 mg/dl.

Examining the association between depressive symptoms and the Life’s Simple 7 may lead to a better understanding of the mechanisms linking depressive symptoms with cardiovascular prognosis and may inform public health efforts to improve cardiovascular disease among vulnerable populations with psychological distress. We therefore compared the prevalence of ideal, intermediate, and poor levels of Life’s Simple 7 components and summary scores of health behaviors and biological measures on the Life’s Simple 7 among participants in the Reasons for Geographic and Racial Differences in Stroke (REGARDS) study with and without depressive symptoms. The REGARDS study provides a broad sample of US adults from which to examine this association and has a sufficient sample size to test whether depressive symptoms are differentially associated with individual components of cardiovascular health.

## Methods

### Ethics Statement

The organization of REGARDS is comprised of an Operations Center and Survey Research Unit at the University of Alabama at Birmingham, a Central Laboratory at the University of Vermont, an Electrocardiogram Reading Center at Wake Forest University, an in-home exam component provided by Examination Management Services Inc. (EMSI), and a medical monitoring and stroke adjudication center at Alabama Neurological Institute Inc. Additional oversight for the study is provided by the National Institute of Neurological Disorders and Stroke (NINDS). Study methods were reviewed and approved by the Institutional Review Boards of each of these organizations and by an external observational study monitoring board appointed by the funding agency, NINDS. As REGARDS participants were first contacted by telephone, participants initially provided verbal informed consent. At subsequent in-home study visits, trained EMSI personnel reviewed and obtained written informed consent from the participants.

### Study Participants

Between January 2003 and October 2007, 30,239 white and African American US adults, ≥45 years of age were enrolled into the REGARDS study, a population-based cohort study of stroke incidence and cognitive decline.^12^ African Americans and residents from 8 Southern US states that comprise the “stroke buckle” (coastal plain region of North Carolina, South Carolina, and Georgia) and the “stroke belt” (remainder of North Carolina, South Carolina, and Georgia, plus Alabama, Mississippi, Tennessee, Arkansas, and Louisiana).were oversampled for inclusion. Potential participants were identified from commercially available lists of US residents and were recruited through an initial mailing followed by telephone contacts. The response rate of 33% and the cooperation rate of 49% were similar to those obtained in other large epidemiologic studies. [23].

### Data Collection

Data were collected via computer-assisted telephone interviews (CATI) and in-home study visits. Data collected using the CATI included sociodemographics, medical history, depressive symptoms, cigarette smoking, and physical activity. Data collected during in-home study visits included a blood sample, electrocardiogram, and pill bottle review to confirm medications. Participants who reported a history of myocardial infarction (MI), coronary revascularization, or evidence of prior MI on the in-home electrocardiogram were classified as having coronary heart disease (CHD). Food intake was assessed by asking participants to self-administer the Block 98 Food Frequency Questionnaire (FFQ) [24], [25] and to mail completed forms to the REGARDS coordinating center. Nutrient analysis was conducted by NutritionQuest (www.nutritionquest.com).

### Life’s Simple 7

Components of the Life’s Simple 7 include cigarette smoking, physical activity, diet, BMI, blood pressure, cholesterol and glucose (Table 1).^7^ Definitions for the physical activity and glucose components were modified from those proposed by Lloyd-Jones et al. [20] to fit the REGARDS data collection instrument. Specifically, instead of defining ideal physical activity as ≥150 minutes/week of moderate intensity activity or ≥70 minutes/week of vigorous activity, participants were asked “How many times per week do you engage in intense physical activity, enough to work up a sweat?” and were classified as ideal, intermediate, and poor if they performed intense physical activity ≥4, 1 to 3, and 0 times per week, respectively. As not all participants fasted prior to the study visit (n = 2,378), the following cut-points for non-fasting glucose were added to the glucose definition to retain the maximum amount of data for the glucose component: ideal <140 mg/dl; intermediate 140–199 mg/dl or treated to <140 mg/dl; poor ≥200 mg/dl. [26] Smoking status was determined by three questions: “Have you smoked at least 100 cigarettes in your lifetime?”, “Do you smoke cigarettes now, even occasionally?”, and “How old were you when you stopped smoking?” Participants who reported never smoking more than 100 cigarettes in their lifetime or who quit smoking more than 12 months ago were categorized as ideal on the smoking component; participants who had smoked more than 100 cigarettes in their lifetime, but had stopped smoking ≤12 months ago were categorized as intermediate, and participants who had smoked >100 cigarettes in their lifetime and were actively smoking were categorized as poor. BMI was calculated using height and weight as measured during the in-home study visit. Diet was categorized based on responses to the FFQ. Specifically, diet categorization depended on the number of the following criteria that were met: fish consumption ≥2 servings/week, fruit/vegetables ≥4.5 cups/day, sodium intake <1500 mg/day, sugar <450 kcal/week, and fiber/carbohydrate ratio >0.1. Blood pressure was measured as the average of two systolic and diastolic blood pressure measurements obtained using a standardized protocol. Total cholesterol was measured using an enzymatic reaction.

### Depressive Symptoms

The 4-item Centers for Epidemiologic Studies of Depression (CESD-4) scale was used to assess depressive symptoms. [27] The scale is comprised of items which assess how many days in the prior week participants felt depressed, felt lonely, had crying spells, and felt sad. Response options for each item include: less than 1 day (0 points), 1–2 days (1 point), 3–4 days (2 points), and 5–7 days (3 points). Each item is scored individually and then items are summed such that the total score can range from 0 to 12 points. Participants with a CESD-4 score ≥4 points were categorized as having depressive symptoms. This cut-point has 79% sensitivity and 86% specificity for identifying clinically significant depressive symptoms as measured by the full 20-item CESD scale (i.e., CESD-20 scores ≥16). [28] Participants were also categorized into three groups of increasing depressive symptom severity: minimal depressive symptoms (CESD-4 of <4); mild-moderate depressive symptoms (CESD-4 of 4 to <8); and moderate-severe depressive symptoms (CESD-4 of ≥8).

### Statistical Analyses

Participants were excluded from the present analysis if they had missing information on one or more Life’s Simple 7 components (n = 9,931). The most common component with missing information, accounting for 85% of those excluded, was diet as many participants did not return the FFQ. An additional 215 participants were excluded because they did not complete the CESD-4. After these exclusions, there were 20,093 participants with data for the analyses presented below. Those excluded due to missing information were similar to those included with respect to mean age (65 years) and gender (54% women). Those excluded were more likely to be African American (58%) and to have depressive symptoms (14%).

Demographic and cardiovascular health characteristics were compared for REGARDS study participants with and without depressive symptoms using t-tests and chi-square tests, as appropriate. Prevalence ratios for having an ideal level of each Life’s Simple 7 component in those with compared to those without depressive symptoms were calculated using Poisson regression models with robust standard error estimates. [29] Also, prevalence ratios for having ≥2, ≥3, ≥4 and ≥5 ideal components in those with versus without depressive symptoms were calculated. Finally, prevalence ratios for having poor levels of each Life’s Simple 7 component were calculated. Prevalence ratios were adjusted for age, race, sex, geographic region of residence, income, and education.

Each Life’s Simple 7 component was also assigned a score of 1, 2, or 3 points to represent poor, intermediate, or ideal health, respectively. The assignment to one of these 3 categories was made in accordance with the recommendations of the developers of the Life’s Simple 7 (Table 1). The points were summed such that the total Life’s Simple 7 score could range from 7 (all components poor) to 21 (all components ideal). Additionally, sub-scores were summed separately for the 4 health behaviors and the 3 biological measures. T-tests were used to compare total Life Simple 7 scores and sub-scores among individuals with and without depressive symptoms. Additionally, mean differences in these scores were compared between individuals with and without depressive symptoms after adjustment for age, race, sex, geographic region of residence, education and income using linear regression models. To determine if a graded association was present between severity of depressive symptoms and Life’s Simple 7 scores, adjusted differences in the scores were calculated for participants with CESD-4 scores of <4, 4 to <8 and ≥8. To determine whether antidepressant use influenced Life’s Simple 7 scores, Life’s Simple 7 scores were compared among participants taking and not taking antidepressants. Finally, to test whether the association between depressive symptoms and Life’s Simple 7 scores differed by CHD status a multiplicative interaction term (CHD * depressive symptoms) was included in the model. Analyses were conducted using SAS 9.2 (SAS Institute, Cary, NC).

## Results

### Participant Characteristics

Participants in this analysis had a mean age of 65 years, 56% were women, and 33% were African American. The prevalence of participants with elevated depressive symptoms in the sample was 9.8%. Compared to those without depressive symptoms, participants with depressive symptoms were younger, more likely to be women, and more likely to be African American, live in the “stroke belt” or “stroke buckle”, have less than high school education, and have a household income <$20,000 per year (Table 2). Additionally, those with depressive symptoms were more likely to be taking medications for hyperlipidemia, diabetes, and hypertension, and they had a higher prevalence of CHD (Table 2).

**Table 2:** Characteristics of REGARDS Study Participants With and Without Depressive Symptoms.

Characteristic^a^	No Depressive Symptoms(n = 18,134)	Depressive Symptoms^b^(n = 1,959)	p-value
Age, years	65.1 (9.2)	62.6 (9.5)	<0.001
African American, %	32.0	43.9	<0.001
Women, %	53.9	70.7	<0.001
Geographic region, %			
Stroke belt	34.2	37.7	<0.001
Stroke buckle	21.6	23.8	
Other	44.2	38.5	
Less than high school education, %	8.5	19.2	<0.001
Annual household income <$20,000, %	15.4	37.9	<0.001
History of coronary heart disease, %	16.5	21.2	<0.001
Medication use, %			
Antidiabetes medication use, %	17.5	24.7	<0.001
Lipid-lowering medication use, %	33.9	36.5	0.021
Antihypertensive medication use, %	50.5	58.8	<0.001
Antidepressant medication use, %	10.9	26.5	<0.001
Health behaviors			
Current smoker, %	12.6	23.8	<0.001
Obese (body mass index ≥30 kg/m^2^), %	35.3	44.9	<0.001
Physical activity <1 time per week, %	31.0	46.3	<0.001
Diet factors			
Fish consumption, grams per week	26.0 (31.4)	26.9 (33.9)	0.306
Sodium intake, mg per day	2264 (1053)	2429 (1188)	<0.001
Sugar, mg per day	261.7 (217.7)	306.2 (256.9)	<0.001
Fruits and vegetables, cups per week	4.4 (2.7)	4.0 (2.6)	<0.001
Fiber/carbohydrate intake ratio	0.082 (0.031)	0.073 (0.029)	<0.001
Biological measures			
Total cholesterol, mg/dL	191.8 (39.2)	195.4 (43.1)	<0.001
Systolic blood pressure, mmHg	126.6 (16.1)	127.6 (17.7)	0.017
Diastolic blood pressure, mmHg	76.1 (9.4)	76.7 (10.3)	0.007
Fasting serum glucose^c^, mg/dL	99.9 (27.7)	104.9 (36.8)	<0.001

a Data expressed as mean (SD) unless otherwise specified.

b Depressive symptoms were defined as a score ≥4 on the 4-item Center for Epidemiologic.

Studies Depression Scale.

c Among 17,715 participants who fasted overnight prior to their REGARDS in-home study visit.

### Depressive Symptoms and Cardiovascular Health

**Figure 1:**
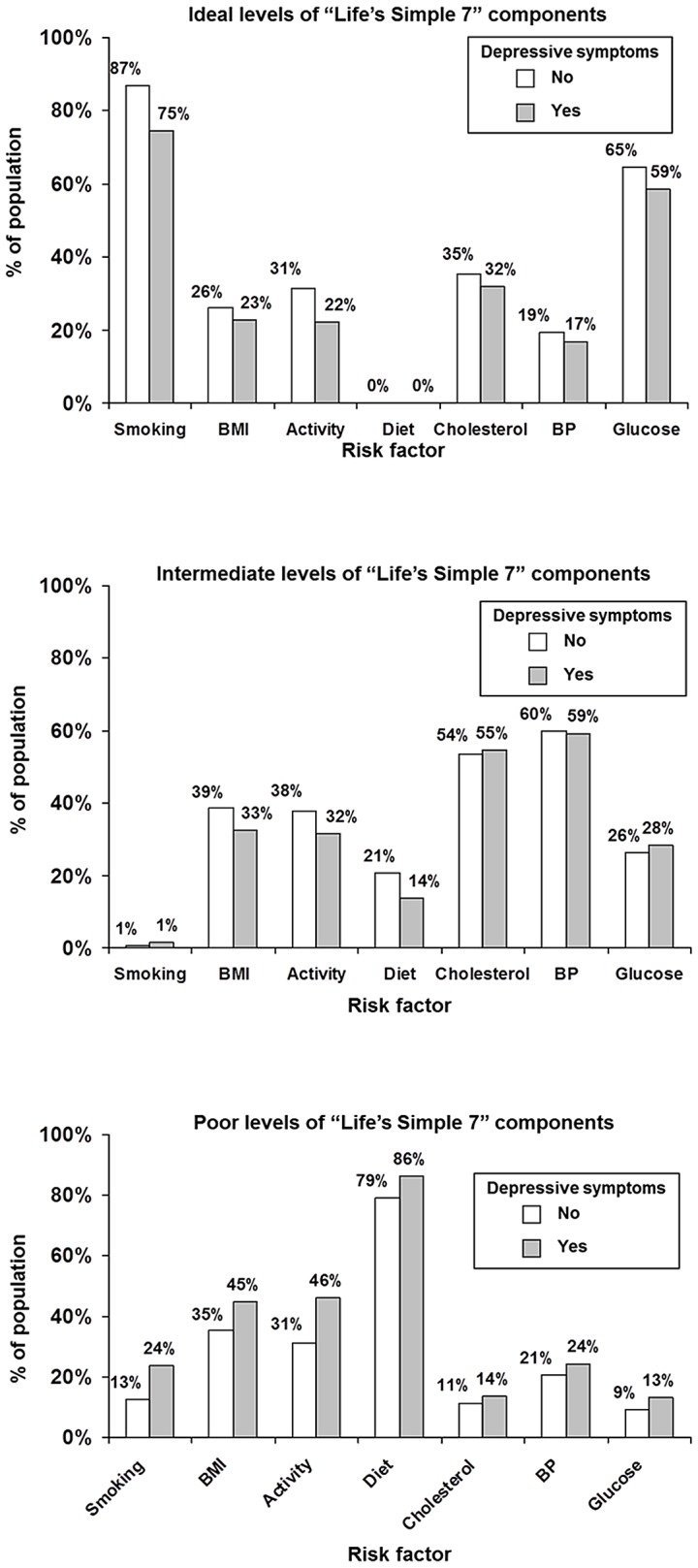
Distribution of Life’s Simple 7 Components as Ideal, Intermediate and Poor Among REGARDS Participants With and Without Elevated Depressive Symptoms^a^. ^a^Depressive symptoms were defined as a score ≥4 on the 4-item Center for Epidemiologic Studies Depression Scale.

**Figure 2:**
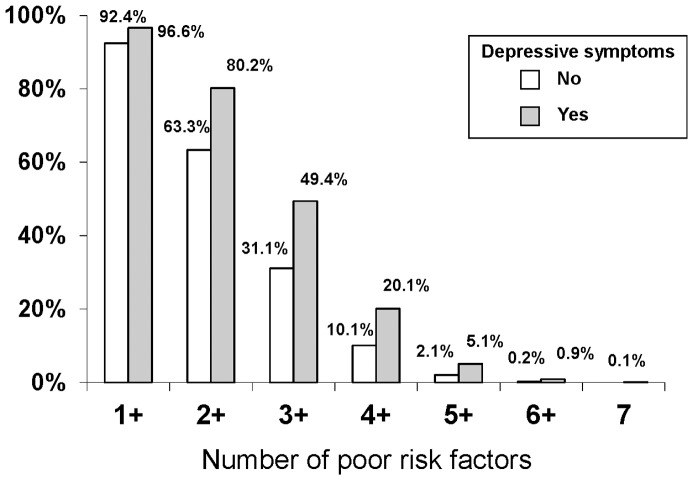
Number of Poor Life’s Simple 7 Components for REGARDS Study Participants with and without Depressive Symptoms.

Participants with depressive symptoms were more likely to have poor levels of each component (Figure 1) and these differences were significant for each component other than cholesterol on adjusted comparisons (Table 3). These associations were strongest for smoking and physical activity; participants with elevated depressive symptoms were 41% more likely to be active smokers and 38% more likely to not be performing any intense physical activity. Participants with depressive symptoms were also more likely to have multiple Life’s Simple 7 components in the poor range (Figure 2). Conversely, participants with depressive symptoms were less likely to have ideal levels of each component in unadjusted comparisons (Figure 1). These differences were significant (p<0.05) after adjustment for each component other than diet and BMI with adjusted prevalence ratios (95% CI) ranging from 0.76 (0.70–0.83) for ideal physical activity to 0.93 (0.89–0.97) for glucose. Of note, no participants met the ideal criteria for diet.

**Table 3:** Adjusted Prevalence Ratios for Poor Levels of Life’s Simple 7 Components for Participants With Compared to those Without Depressive Symptoms*.

Life’s Simple 7 Component at Poor Level	Prevalence Ratios(95% Confidence Interval)	P-Value
Smoking (Current)	1.41 (1.29–1.55)	<0.001
Physical Activity (none)	1.38 (1.31–1.46)	<0.001
Diet (<2 out of 5 healthy diet criteria)	1.08 (1.06–1.10)	<0.001
Body mass index (BMI) (BMI ≥30 kg/m^2^)	1.09 (1.04–1.15)	0.001
Cholesterol (≥240 mg/dL)	1.05 (0.93–1.18)	0.436
Blood pressure (systolic/diastolic ≥140/≥80 mmHg)	1.11 (1.02–1.11)	0.012
Glucose (fasting ≥126 mg/dL or non-fasting ≥200 mg/dL)	1.24 (1.09–1.41)	<0.001

* Prevalence ratios are for having poor levels versus intermediate or ideal levels of Life’s Simple 7 components comparing those with versus those without depressive symptoms. Prevalence ratios are adjusted for age, race, sex, geographic region of residence, education and income. Depressive symptoms are defined as a score ≥4 on the 4-item Center for Epidemiologic Studies Depression Scale.

Participants with depressive symptoms had lower scores (i.e., worse cardiovascular health) on the total Life’s Simple 7 and on the health behavior and biological measures sub-scales (Table 4). Also, there was a graded association between increasing depressive symptom severity and lower Life’s Simple 7 scores (Table 5).

**Table 4:** Life’s Simple 7 Total Score, and Health Behavior and Biological Factor Subscores and Adjusted Differences in Scores Among Participants With Versus Without Depressive Symptoms.

Scale	No Depressive Symptoms(n = 18,134)	Depressive Symptoms^b^(n = 1,959)	P-Value
Life’s Simple 7 Scale (7-Item; 7–21 points)			
Total Score, mean (SD)	14.6 (2.0)	13.7 (2.1)	<0.001
Adjusted difference^a^, mean (SE)	0 (ref)	−0.63 (0.05)	<0.001
Health Behavior Subscale (4-Item; 4–12 points)			
Subscore, mean (SD)	7.9 (1.4)	7.2 (1.4)	<0.001
Adjusted difference, mean (SE)	0 (ref)	−0.47 (0.03)	<0.001
Biological Measures Subscale (3-Item; 3–9 points)			
Subscore, mean (SD)	6.8 (1.2)	6.6 (1.3)	<0.001
Adjusted difference^†^, mean (SE)	0 (ref)	−0.15 (0.03)	<0.001

Abbreviations: SD, standard deviation; SE, standard error.

a Scores were adjusted for age, race, sex, geographic region of residence, education, and income.

b Depressive symptoms were defined as a score ≥4 on the 4-item Center for Epidemiologic.

Studies Depression Scale.

**Table 5:** Graded Association Between Life’s Simple 7 Total Score and Subscores According to Depressive Symptom Severity.

Scale	CESD <4(n = 18,134)	CESD 4 to <8(n = 1,521)	CESD ≥8(n = 438)
Life’s Simple 7 Scale (7-Item; 7–21 points)			
Total Score, mean (SD)	14.6 (2.0)	13.9 (2.1)	13.3 (2.1)
Adjusted difference^a^, mean (SE)	0 (ref)	−0.54 (0.05)	−0.95 (0.10)
Health Behavior Subscale (4-Item; 4–12 points)			
Subscore, mean (SD)	7.9 (1.4)	7.3 (1.4)	6.9 (1.4)
Adjusted difference^a^, mean (SE)	0 (ref)	−0.42 (0.04)	−0.66 (0.07)
Biological Measures Subscale (3-Item; 3–9 points)			
Subscore, mean (SD)	6.8 (1.2)	6.6 (1.3)	6.4 (1.3)
Adjusted difference^a^ mean (SE)	0 (ref)	−0.12 (0.03)	−0.28 (0.06)

Abbreviations: CESD, 4-item Center for Epidemiologic Studies Depression Scale; SD, standard deviation; SE, standard error.

a Scores were adjusted for age, race, sex, geographic region of residence, education, and income.

There was a larger difference in scores between participants with and without depressive symptoms on the health behavior sub-scales than on the biological sub-scales. This difference was larger than could be expected solely as a result of their being one fewer item on the biological sub-scale. The finding was more prominent when comparing those with more severe depressive symptoms (CESD-4≥8) to those without depressive symptoms (CESD-4<4). For this comparison, there was a 1 point difference between depressed and non-depressed participants on the health behavior sub-scale as compared to only a 0.4 point difference on the biological measure sub-scale (Table 5).

### Sensitivity Analyses

Depressive symptoms were associated with lower Life’s Simple 7 scores irrespective of whether participants were taking antidepressant medications. In the subgroup of participants with depressive symptoms, those taking antidepressants had lower Life’s Simple 7 scores than those not taking antidepressants (13.3 points versus 13.9 points, respectively; p<0.001). Similarly, in the subgroup of participants without depressive symptoms, those who were taking antidepressants had lower Life’s Simple 7 scores (14.2 points versus 14.7 points; p<0.001).

Finally, depressive symptoms were associated with lower Life’s Simple 7 scores in both those with and without pre-existing CHD. The adjusted difference in Life’s Simple 7 score between those with and without depressive symptoms among those with CHD was −0.83 (SD 0.11) as compared to −0.68 (SD 0.05) among those without CHD (p-value for interaction = 0.056).

## Discussion

The current analyses demonstrate a strong and graded association between depressive symptoms and worse cardiovascular health as measured by the AHA’s Life’s Simple 7. These associations were present for the total Life’s Simple 7 score and for having poor and ideal levels of the majority of the individual components of the metric that represent health behaviors (i.e., cigarette smoking, physical activity) and biological measures of cardiovascular health other than cholesterol (i.e., blood pressure and glucose).

Depressive symptoms were associated with worse levels on both behavioral and biological components of the Life’s Simple 7. The strongest associations were present for smoking and physical activity: those with depressive symptoms were about 41% more likely to be active smokers and to be 38% more likely to forego regular intense physical activity. In contrast, those with depressive symptoms were not more likely to have poor levels of total cholesterol and were only modestly more likely to have poor levels of blood pressure and glucose (24% and 11%, respectively). While a direct comparison between sub-scores summarizing biological and behavioral components should be made with caution given the differing number of items in each sub-score, the finding of a stronger relationship between depressive symptoms and health behavior is consistent with prior studies showing that the impact of psychological distress on cardiovascular risk is more strongly associated with behavioral factors than biological ones. [30,31 Our study extends these finding by comprehensively assessing these relationships in a large, diverse, population-based sample.

Depressive symptoms were common in the REGARDS population; the 9.8% prevalence in REGARDS is similar to the 9% prevalence of combined major or “other” depression measured among adults ≥45 years old in a recent survey of the US population using a different depression measure. [32] Accordingly, the impact of depressive symptoms on cardiovascular health extends to a sizable portion of the population.

The association between depressive symptoms and Life’s Simple 7 score was present in participants with and without preexisting CHD. This is consistent with prior literature showing that depressed patients have lower adherence to recommended health behaviors before and after incident cardiac events. [8], [33] Hence, it will be important to consider the impact of depressive symptoms on adherence for both primary and secondary cardiovascular prevention.

The use of antidepressants was associated with lower Life’s Simple 7 scores (i.e., worse cardiovascular health) among participants with depressive symptoms. The presence of depressive symptoms despite taking antidepressant medications may be indicative of treatment-resistant depression. [34] Accordingly, our results suggest that treatment-resistant depression may be an important marker of poor cardiovascular health, and individuals with this condition may represent a vulnerable group in especially high need of interventions to improve their cardiovascular health. An alternative hypothesis is that antidepressant medications may exert direct adverse effects on health behaviors and biological factors. For example, some antidepressants have been associated with weight gain [35] and blood pressure changes. [36] Additional research into the potentially adverse cardiovascular effects of antidepressants is warranted.

There are several limitations to the current analysis. The cross-sectional and observational nature of these data prevent us from ascribing causal attributions to depressive symptoms on cardiovascular health. The CESD-4 measures depressive symptoms rather than clinical depression and the extrapolation of our results to patients with clinical depression must be made with caution. Nevertheless, elevated scores on the CESD-4 have been validated as a proxy for clinical depression [27] and the prevalence of depressive symptoms by the CESD-4 in our sample was similar to the prevalence of depression measured in another population sample using a different scale. [32] Approximately one-third of participants were not included due to missing data on the Life’s Simple 7. As participants with missing data were more likely to have depressive symptoms, we may have underestimated the true prevalence of depressive symptoms in this cohort. Further, we had insufficient information to precisely replicate Life’s Simple 7 metrics for physical activity and glucose. Antidepressants may have been prescribed for indications other than depression, limiting the interpretability of the association between antidepressant use and Life’s Simple 7 scores. Finally, REGARDS recruitment oversampled individuals who were African American or lived in the stroke belt and participants. Nevertheless, our main results were adjusted for geographic region and race.

### Implications

Our analysis showing worse cardiovascular health according to the Life’s Simple 7 metric amongst those with depressive symptoms highlights that depressed individuals are a vulnerable group in need of special consideration to attain optimal cardiovascular health on both health behaviors and biological risks markers. Given the high prevalence of elevated depressive symptoms in the population, public health efforts to achieve the AHA 2020 goals of improving cardiovascular health by 20% by 2020 will need to consider approaches to screen for and provide behavior modification for depressed individuals. Enhanced depression care alone has not consistently resulted in significant improvements in cardiovascular health behaviors or risk factor control even when depression was reduced. [37], [38] However, enhanced depression care that is paired with treatment to reach biological factor goals using a collaborative care approach has been successful at improving risk factor control in at least one major study. [39], [40] Additional research is needed to identify the best approaches to achieve ideal cardiovascular health in depressed individuals.

## References

[1] Nicholson A, Kuper H, Hemingway H (2006) Depression as an aetiologic and prognostic factor in coronary heart disease: a meta-analysis of 6362 events among 146 538 participants in 54 observational studies. Eur Heart J 27: 2763–2774. 17082208 10.1093/eurheartj/ehl338

[2] Glymour MM, Maselko J, Gilman SE, Patton KK, Avendano M (2010) Depressive symptoms predict incident stroke independently of memory impairments. Neurology 75: 2063–2070. 21135381 10.1212/WNL.0b013e318200d70e PMC2995534

[3] Pan A, Okereke OI, Sun Q, Logroscino G, Manson JE, et al.. (2011) Depression and Incident Stroke in Women. Stroke. 10.1161/STROKEAHA.111.617043 PMC3183155 21836097

[4] van Melle JP, de Jonge P, Spijkerman TA, Tijssen JG, Ormel J, et al (2004) Prognostic association of depression following myocardial infarction with mortality and cardiovascular events: a meta-analysis. Psychosomatic Medicine 66: 814–822. 15564344 10.1097/01.psy.0000146294.82810.9c

[5] Wassertheil-Smoller S, Shumaker S, Ockene J, Talavera GA, Greenland P, et al (2004) Depression and cardiovascular sequelae in postmenopausal women. The Women's Health Initiative (WHI). Arch Intern Med 164: 289–298. 14769624 10.1001/archinte.164.3.289

[6] Joynt KE, Whellan DJ, O'Connor CM (2003) Depression and cardiovascular disease: mechanisms of interaction. Biol Psychiatry 54: 248–261. 12893101 10.1016/s0006-3223(03)00568-7

[7] Rieckmann N, Kronish IM, Haas D, Gerin W, Chaplin WF, et al (2006) Persistent depressive symptoms lower aspirin adherence after acute coronary syndromes. Am Heart J 152: 922–927. 17070160 10.1016/j.ahj.2006.05.014

[8] Kronish IM, Rieckmann N, Halm EA, Shimbo D, Vorchheimer D, et al (2006) Persistent depression affects adherence to secondary prevention behaviors after acute coronary syndromes. J Gen Intern Med 21: 1178–1183. 16899061 10.1111/j.1525-1497.2006.00586.x PMC1831650

[9] DiMatteo MR, Lepper HS, Croghan TW (2000) Depression is a risk factor for noncompliance with medical treatment: meta-analysis of the effects of anxiety and depression on patient adherence. Arch Intern Med 160: 2101–2107. 10904452 10.1001/archinte.160.14.2101

[10] Thorndike AN, Regan S, McKool K, Pasternak RC, Swartz S, et al (2008) Depressive symptoms and smoking cessation after hospitalization for cardiovascular disease. Arch Intern Med 168: 186–191. 18227366 10.1001/archinternmed.2007.60

[11] Rutledge T, Hogan BE (2002) A quantitative review of prospective evidence linking psychological factors with hypertension development. Psychosom Med 64: 758–766. 12271106 10.1097/01.psy.0000031578.42041.1c

[12] Campayo A, de Jonge P, Roy JF, Saz P, de la Camara C, et al (2010) Depressive disorder and incident diabetes mellitus: the effect of characteristics of depression. Am J Psychiatry 167: 580–588. 20123914 10.1176/appi.ajp.2009.09010038

[13] Delaney JA, Oddson BE, Kramer H, Shea S, Psaty BM, et al (2010) Baseline depressive symptoms are not associated with clinically important levels of incident hypertension during two years of follow-up: the multi-ethnic study of atherosclerosis. Hypertension 55: 408–414. 20065156 10.1161/HYPERTENSIONAHA.109.139824 PMC2821214

[14] Pan A, Lucas M, Sun Q, van Dam RM, Franco OH, et al (2010) Bidirectional association between depression and type 2 diabetes mellitus in women. Arch Intern Med 170: 1884–1891. 21098346 10.1001/archinternmed.2010.356 PMC3065781

[15] Katon WJ, Lin EH, Russo J, Von Korff M, Ciechanowski P, et al (2004) Cardiac risk factors in patients with diabetes mellitus and major depression. J Gen Intern Med 19: 1192–1199. 15610329 10.1111/j.1525-1497.2004.30405.x PMC1492592

[16] Lustman PJ, Anderson RJ, Freedland KE, de Groot M, Carney RM, et al (2000) Depression and poor glycemic control: a meta-analytic review of the literature. Diabetes Care 23: 934–942. 10895843 10.2337/diacare.23.7.934

[17] Licht CM, de Geus EJ, Seldenrijk A, van Hout HP, Zitman FG, et al (2009) Depression is associated with decreased blood pressure, but antidepressant use increases the risk for hypertension. Hypertension 53: 631–638. 19237679 10.1161/HYPERTENSIONAHA.108.126698

[18] Yan LL, Liu K, Matthews KA, Daviglus ML, Ferguson TF, et al (2003) Psychosocial factors and risk of hypertension: the Coronary Artery Risk Development in Young Adults (CARDIA) study. JAMA 290: 2138–2148. 14570949 10.1001/jama.290.16.2138

[19] Tedders SH, Fokong KD, McKenzie LE, Wesley C, Yu L, et al.. (2011) Low cholesterol is associated with depression among US household population. J Affect Disord. 10.1016/j.jad.2011.06.045 21802743

[20] Lloyd-Jones DM, Hong Y, Labarthe D, Mozaffarian D, Appel LJ, et al (2010) Defining and setting national goals for cardiovascular health promotion and disease reduction: the American Heart Association's strategic Impact Goal through 2020 and beyond. Circulation 121: 586–613. 20089546 10.1161/CIRCULATIONAHA.109.192703

[21] Folsom AR, Yatsuya H, Nettleton JA, Lutsey PL, Cushman M, et al (2011) Community prevalence of ideal cardiovascular health, by the American Heart Association definition, and relationship with cardiovascular disease incidence. J Am Coll Cardiol 57: 1690–1696. 21492767 10.1016/j.jacc.2010.11.041 PMC3093047

[22] Ford ES, Greenlund KJ, Hong Y (2012) Ideal cardiovascular health and mortality from all causes and diseases of the circulatory system among adults in the United States. Circulation 125: 987–995. 22291126 10.1161/CIRCULATIONAHA.111.049122 PMC4556343

[23] Jackson R, Chambless LE, Yang K, Byrne T, Watson R, et al (1996) Differences between respondents and nonrespondents in a multicenter community-based study vary by gender ethnicity. The Atherosclerosis Risk in Communities (ARIC) Study Investigators. J Clin Epidemiol 49: 1441–1446. 8970495 10.1016/0895-4356(95)00047-x

[24] Block G, Woods M, Potosky A, Clifford C (1990) Validation of a self-administered diet history questionnaire using multiple diet records. J Clin Epidemiol 43: 1327–1335. 2254769 10.1016/0895-4356(90)90099-b

[25] Block G, Hartman AM, Naughton D (1990) A reduced dietary questionnaire: development and validation. Epidemiology 1: 58–64. 2081241 10.1097/00001648-199001000-00013

[26] American Diabetes A (2011) Standards of medical care in diabetes–2011. Diabetes Care 34 Suppl 1S11–61. 21193625 10.2337/dc11-S011 PMC3006050

[27] Melchior LA, Huba GJ, Brown VB, Reback CJ (1993) A Short Depression Index for Women. Educational and Psychological Measurement 53: 1117–1125.

[28] Radloff L (1977) The CES-D Scale: a self-report depression scale for research in the general population. Appl Psychol Meas 1: 385–401.

[29] Behrens T, Taeger D, Wellmann J, Keil U (2004) Different methods to calculate effect estimates in cross-sectional studies - A comparison between prevalence odds ratio and prevalence ratio. Methods of Information in Medicine 43: 505–509. 15702210

[30] Hamer M, Molloy GJ, Stamatakis E (2008) Psychological distress as a risk factor for cardiovascular events: pathophysiological and behavioral mechanisms. J Am Coll Cardiol 52: 2156–2162. 19095133 10.1016/j.jacc.2008.08.057

[31] Whooley MA, de Jonge P, Vittinghoff E, Otte C, Moos R, et al (2008) Depressive symptoms, health behaviors, and risk of cardiovascular events in patients with coronary heart disease. JAMA 300: 2379–2388. 19033588 10.1001/jama.2008.711 PMC2677371

[32] Current depression among adults–United States, 2006 and 2008. MMWR Morb Mortal Wkly Rep 59: 1229–1235. 20881934

[33] Ziegelstein RC, Fauerbach JA, Stevens SS, Romanelli J, Richter DP, et al (2000) Patients with depression are less likely to follow recommendations to reduce cardiac risk during recovery from a myocardial infarction. Arch Intern Med 160: 1818–1823. 10871976 10.1001/archinte.160.12.1818

[34] Rush AJ, Thase ME, Dube S (2003) Research issues in the study of difficult-to-treat depression. Biol Psychiatry 53: 743–753. 12706958 10.1016/s0006-3223(03)00088-x

[35] Serretti A, Mandelli L (2010) Antidepressants and body weight: a comprehensive review and meta-analysis. J Clin Psychiatry 71: 1259–1272. 21062615 10.4088/JCP.09r05346blu

[36] Feighner JP (1995) Cardiovascular safety in depressed patients: focus on venlafaxine. J Clin Psychiatry 56: 574–579. 8530334

[37] Lin EH, Katon W, Rutter C, Simon GE, Ludman EJ, et al (2006) Effects of enhanced depression treatment on diabetes self-care. Ann Fam Med 4: 46–53. 16449396 10.1370/afm.423 PMC1466986

[38] Huffman JC, Mastromauro CA, Sowden G, Fricchione GL, Healy BC, et al (2011) Impact of a depression care management program for hospitalized cardiac patients. Circ Cardiovasc Qual Outcomes 4: 198–205. 21386067 10.1161/CIRCOUTCOMES.110.959379

[39] Katon W, Lin EH, Von Korff M, Ciechanowski P, Ludman E, et al (2010) Integrating depression and chronic disease care among patients with diabetes and/or coronary heart disease: the design of the TEAMcare study. Contemp Clin Trials 31: 312–322. 20350619 10.1016/j.cct.2010.03.009 PMC3726010

[40] Katon WJ, Lin EH, Von Korff M, Ciechanowski P, Ludman EJ, et al (2010) Collaborative care for patients with depression and chronic illnesses. N Engl J Med 363: 2611–2620. 21190455 10.1056/NEJMoa1003955 PMC3312811

